# Effects of Selenium and Melatonin on Ocular Ischemic Syndrome

**DOI:** 10.1155/2019/8080564

**Published:** 2019-12-06

**Authors:** Hande Hüsniye Telek

**Affiliations:** Beytepe Murat Erdi Eker State Hospital, Ahlatlıbel District, 06640 Beytepe Çankaya, 1746. St., 06800 Çankaya/Ankara, Turkey

## Abstract

**Purpose:**

To determine the effects of selenium, melatonin, and selenium + melatonin administered for one month on anterior chamber (AC) malondialdehyde (MDA) and AC glutathione (GSH) levels in patients with ocular ischemic syndrome.

**Materials and Methods:**

Thirty-five patients were included in the study. Study groups were formed as follows: (1) control group, (2) ischemia group, (3) selenium + ischemia group, (4) melatonin + ischemia group, and (5) selenium + melatonin + ischemia group. AC samples were obtained. MDA and GSH levels in AC samples were evaluated.

**Results:**

MDA levels were significantly increased in ischemia groups. Selenium and melatonin supplementation resulted in reduction of MDA levels and significant increase in GSH values.

**Discussion:**

Increased lipid peroxidation associated with ischemia of the anterior segment has been prevented by selenium and melatonin supplementation. This trial is registered with ClinicalTrials.gov NCT04005222.

## 1. Introduction

Ocular ischemic syndrome (OIS) comprises a spectrum of ocular characteristics caused by arterial hypoperfusion of the eye. The condition manifests visual deterioration, pain, and diverse signs of both the anterior and the posterior segments as well as abnormalities in other ophthalmic artery supplied orbital structures. The main cause of OIS is carotid artery stenosis [1]. Many OIS patients are usually undiagnosed or misdiagnosed by ophthalmologists due to the asymptomatic onset and complicated ocular manifestations. Such failures can result in irreversible blindness and an increased mortality rate [2, 3].

Ischemia is a condition that develops as a result of inadequate blood supply to the organ or tissue. It causes reversible or irreversible tissue or organ damage [4]. Reperfusion is described as restoration of blood flow or oxygenation of tissues or organs; damage that occurs to tissues or organs during reperfusion following ischemia is defined as reperfusion damage. Although multiple mechanisms are involved in reperfusion damage, the primarily accused mechanism is rapid generation of free radical derivatives with intracellular molecular oxygen influx [5].

Reperfusion is the prerequisite for recovery from ischemia. However, paradoxically, reintroduction of molecular oxygen with reperfusion generates harmful oxygen radicals associated with lipid peroxidation and causes loss of cell membrane integrity [6]. Providing reperfusion to ischemic tissues may lead to increased cell damage [4]. Free radicals are unstable, nondurable, and extremely reactive molecules that contain one or more unpaired electrons in the outer orbit. They attack and damage other cells to stabilize [7]. Free radicals that are classified as reactive oxygen species (ROS) or reactive nitrogen species (RNS) are continuously generated during aerobic respiration, cell metabolism, and defense against pathogens [8]. Normally free radicals and antioxidant system are in a state of equilibrium. Increased free radicals or reduced antioxidants impair this balance. If the amount of free radicals exceeds the buffering capacity of available antioxidants, this leads to the development of oxidative stress. These radicals may harm the organism by altering the structure and functions of molecules including proteins, DNA, lipids, and carbohydrates.

Oxidative stress plays an important role in hypertension, diabetes, ischemic conditions, cardiovascular diseases, atherosclerosis, arthritis, cancer, premature ageing, and neurodegenerative diseases such as Alzheimer and Parkinson [9]. A small portion of the oxygen used during oxidative metabolism is transformed to highly reactive and toxic reactive oxygen species. These reactive oxygen species have regulatory roles for some cellular functions at limited concentrations. However, when reactive oxygen species formed exceed the required concentrations, they cause damage to macromolecules including cell organelles, membrane lipids, nuclear and mitochondrial DNA, and proteins [10].

Reactive oxygen species produce lipid peroxidase products, especially malondialdehyde (MDA), through reaction with unsaturated long-chain fatty acids in cell membranes. The resulting lipid peroxidation of free radicals acting on unsaturated fatty acids in the cell membrane can lead to an increase in membrane permeability which may cause cell death [11]. Malondialdehyde (MDA) is a highly reactive and toxic aldehyde which is generated by peroxidation of polyunsaturated fatty acids (PUHA). MDA accumulation can alter membrane permeability and impair viscosity of double-layered lipid membrane. MDA, which is one of the most mutagenic products of lipid peroxidation, is also a biomarker commonly used to evaluate lipid peroxidation [12]. Although substances that react with lipid hydroperoxides, 4-hydroxynoneal isoprostanes, 8-hydroxy-2-deoxyguanosine, malondialdehyde, allantoin, and thiobarbituric acid have been proposed as oxidative markers in our body, malondialdehyde has been the most commonly used marker in recent years [11]. Ischemia-reperfusion damage process based on generation of reactive oxygen species mediated by lipid peroxidation can be measured using products such as MDA [13].

Generation of free radicals, also referred to as reactive oxygen species, has been stabilized by many molecular and enzymatic antioxidants. Examples for molecular antioxidants include vitamins C, A, and E, uric acid, glutathione (GSH), pycnogenol, and thioredoxin; enzymatic antioxidants include catalase (CAT), thioredoxin reductase, glutathione peroxidase (GSHPx), glutathione reductase (GR), glutathione S transferase (GST), ascorbate peroxidase, ascorbate reductase, and glucose-6-phosphate dehydrogenase [14]. Glutathione (GSH) is an antioxidant composed of three amino acids: glutamate, cysteine, and glycine. It is one of the most active antioxidant systems in human physiology [15, 16]. GSH protects the cell and biomolecules from oxidative damage by strong antioxidant activity against reactive oxygen species and reactive nitrogen species [17]. Melatonin, also known as N-acetyl 5-methoxytryptamine, is a potent antioxidant produced by various organs, predominantly the pituitary gland [18, 19]. Melatonin has physiological activities such as circadian rhythm, sleep, and promoting immunity. However, the most important effect of melatonin is antioxidant effect which protects organisms against oxidative stress [20]. Besides direct activity on free radicals, melatonin also shows indirect antioxidant activity by activating certain enzymes including superoxide dismutase, glutathione peroxidase, and catalase [19].

The aim of this study was to determine the effects of selenium and melatonin administered for one month alone or in combination on lipid peroxidation in patients with ocular ischemic syndrome.

## 2. Materials and Methods

In this retroprospective study, patients who presented with the clinical features of OIS or who had a history of OIS and who had visited the Department of Ophthalmology or who were referred by the Department of Cardiology were considered for inclusion. The patients of OIS were included according to the following criteria [21, 22]: (1) when the stenosis of the ipsilateral (to the affected eye) internal carotid artery (ICA) was >50% and the ICA blood flow velocity was abnormal and (2) when there were abnormal ocular symptoms and/or signs that could not be explained by other ocular diseases. The ocular symptoms included amaurosis fugax, visual loss, floaters, metamorphopsia, phosphenes, diplopia, and ocular/periorbital pain. Most patients (88.10%) complained of constitutional symptoms, such as headache, syncope, palpitations, hemiplegia, and claudication. The patients who were suffered from other ocular diseases, including primary glaucoma, uveitis, age-related macular degeneration, symmetrical proliferative diabetic retinopathy, choroidal detachment, retinal detachment, hereditary eye diseases, ocular tumor, or ocular trauma, were excluded. Informed consent was acquired from all of the participants before the collection of clinical materials. The study adhered to the tenets of the Declaration of Helsinki.

All of the OIS patients underwent carotid artery color Doppler imaging (CDI) and/or computed tomographic angiography (CTA) to identify the ICA stenosis. Detailed ophthalmic examinations including best-corrected visual acuity (BCVA), intraocular pressure (IOP), slit-lamp exam, and funduscopy were performed at each follow-up visit. Constitutional and ocular symptoms, medical history (arterial hypertension, diabetes mellitus (DM), hyperlipidemia (HLP), coronary heart disease, cerebrovascular disease, and so on), the clinical department of the first visit, and treatments were also recorded. A statistical description was generated using SPSS for Windows, version 22.0.

Twenty-eight OIS patients were recruited in our study, including 20 males (%71.4) and 8 females (%28.6). The age of onset ranged from 58 to 87 years (65.10 ± 10.95), with the majority of patients aged between 61 and 75 years (69.50%). No statistically significant difference was found for gender and age between groups (*p* < 0.01).

The study was conducted at Trabzon Numune Training and Research Hospital between May 2014 and September 2016. Approval from the Trabzon Numune Training and Research Hospital Ethics Committee was obtained. All procedures performed in studies involving human participants were in accordance with the ethical standards of the institutional and/or national research committee and with the 1964 Helsinki declaration and its later amendments or comparable ethical standards.

Patients were divided into five groups:

1. Control group: seven healthy persons were included in this group. After topical anesthesia, approximately 0.1 cc samples were obtained from the anterior chamber.
2. Ischemia group: seven OIS patients were included in this group. Under topical anesthesia, 0.1 cc samples were obtained from the AC.
3. Selenium + ischemia group: seven OIS patients were included in this group. The patients in this group were supplemented with oral selenium at 0.1 mg doses twice daily for one month. After selenium supplementation period was completed, there was AC sampling as described above.
4. Melatonin + ischemia group: seven OIS patients were included in this group. The patients in this group were supplemented with oral melatonin 3 mg doses twice daily for one month. After supplementation was completed, there was 0.1 cc sampling from AC.
5. Selenium + melatonin + ischemia group: seven OIS patients were included in this group. The patients were supplemented with selenium and melatonin for one month as described above; subsequently, there was 0.1 cc sampling from AC.

In order to determine AC malondialdehyde (MDA) levels, 2.5 ml of 10% TCA (trichloroacetic acid) was put in a test tube, and 0.1 ml of AC sample from the patients was added. Tubes were vortexed and sealed. Incubation was applied for 15 minutes in 90°C water bath. They were cooled in cold water and their absorbance values were read with reference to blank on spectrophotometer at 532 nm. Results were presented as nmol/ml. At the beginning of the testing, blank was prepared by placing the same amount of distilled water instead of plasma in the blank tube and performing the same procedures. In order to measure AC glutathione (GSH) levels, AC samples placed in tubes containing EDTA were centrifuged at 3000 rpm for 5 minutes. The samples were washed 3 times with 0.9% saline solution and 50 *μ*l of each sample was derived. Consecutively, 450 *μ*l of distilled water and 500 *μ*l of 10% sulfosalicylic acid were added. The mixture was cooled in ice for 1 hour and then centrifuged at 4000 rev for 3 minutes. Subsequently, 200 *μ*l of the supernatant was derived and consecutively 8 ml of phosphate buffer with pH 6.8, 78 *μ*l of 1 N NaOH, and 100 *μ*l of Ellman solution were added. After waiting for 5 minutes, absorbance values in reagent tube were read with reference to distilled water on spectrophotometer at 412 nm. Ellman's solution was prepared by dissolving 100 mg of 5′-5′-dithiobis-2-nitrobenzoic acid (DTNB) in 100 ml of pH 7.8 phosphate buffer. Glutathione standard was prepared as 15.34 mg/100 ml by dissolving 15.34 mg of reduced glutathione in 100 ml of 1 nm sodium EDTA. Results were presented as mg/dl.

### 2.1. Statistics

Statistical analysis was performed using SPSS statistics software. Results were described as mean ± standard deviation. Kruskal–Wallis variance analysis was used for intergroup comparisons and Mann–Whitney *U* test was performed for *p* < 0.05. Statistical significance was defined as *p* < 0.05.

## 3. Results

The AC MDA values of study groups were as follows: control group, 0.04 ± 0.01; ischemia group, 0.45 ± 0.08; selenium + ischemia group, 0.06 ± 0.03; melatonin + ischemia group, 0.08 ± 0.05, and selenium + melatonin + ischemia group, 0.10 ± 0.06. Groups were compared and ischemia group was found to have the highest MDA values, and the difference was considered statistically significant (*p* < 0.001). There was no significant difference between the other groups in terms of MDA (*p* values 0.193, 0.496, 0.768, and 0.323, respectively) (Table 1).

The AC GSH values detected in study groups were as follows: control group, 0.42 ± 0.05; ischemia group, 0.15 ± 0.01; selenium + ischemia group, 0.52 ± 0.03; melatonin + ischemia group, 0.48 ± 0.08; and selenium +melatonin + ischemia group, 0.59 ± 0.02. Groups were compared and selenium + melatonin + ischemia group was found to have the highest GSH values while ischemia group showed the lowest GSH values which was statistically significant compared to other groups (*p* < 0.001). Selenium + ischemia group, melatonin + ischemia group, and selenium + melatonin + ischemia groups had higher GSH levels compared to ischemia group. No statistically significant difference was found for AC GSH values between groups except ischemia group (*p* values: 0.231, 0.602, 0.439, and 0.561, respectively) (Table 2).

## 4. Discussion

The ocular ischemic syndrome is a rare condition characterized by chronic ischemia of the anterior and/or posterior segment of the eyes and is primarily caused by the stenosis of the carotid artery. Jingyi Luo et al. have reported that the mortality rate in OIS patients is up to 40% within 5 years of onset. As OIS is usually asymptomatic and has poor outcomes, a strategy for establishing an early diagnosis is essential for saving visual function and improving survival [22]. The major cause of OIS is atherosclerosis [23], and other common causes include giant cell arteritis, thrombogenesis, Takayasu arteritis, trauma, and different types of diseases involving the carotid arteries [24, 25]. The symptoms and clinical signs of OIS are various and nonspecific. Visual loss, orbital pain, and various anterior and posterior segment signs are the most common clinical manifestations in OIS [26]. The visual dysfunction ranged from amaurosis fugax to severe visual loss. The abnormal sensations were primarily present as ocular and/or periorbital pain.

Ischemia-reperfusion can cause serious oxidative damage of tissues in animals [27]. MDA level measurement is among the most commonly used indicators for oxidative stress and tissue damage. For this purpose, MDA levels in AC were determined in our study. In a study conducted by Avci et al. [28], in a rat model developed by 120-minute exposure to ischemia-reperfusion (I/R) of the intrarenal abdominal aorta, the I/R group was found to have increased MDA values. In the study by Gurji et al. [29], increased damage was detected in goats exposed to hind leg ischemia for 90 minutes with a tourniquet and femoral artery clamp and 4-hour reperfusion. Kirisci et al. [30] have reported increased tissue MDA levels in rats exposed to skeletal muscle ischemia-reperfusion for 120 minutes. Tong et al. [27] have also reported increased MDA levels in ischemia-reperfusion.

Selenium, which is highly important for human health, is necessary for a variety of metabolic processes, including thyroid hormone metabolism, protection against oxidative stress, and immunity functions. Selenium supplementation prevents lipid peroxidation. Selenium is a molecule that activates glutathione peroxidase, and, thus, it is involved in the antioxidant mechanisms that prevent oxidant damage [31, 32]. Retinal circadian regulation in both mammals and nonmammalian vertebrates uses melatonin as dark- and light-adaptive neuromodulators. The pineal gland produces and secretes melatonin during the hours of darkness. This neurohormone induces entrainment of the circadian and circannual rhythmicity; it also has many other functions in vertebrates. In high concentrations (micromolar), melatonin is a free radical scavenger. At low levels (picomolar), melatonin acts by binding to membrane receptors [33–35].

The results of our study indicating significantly increased AC MDA levels in OIS patients compared to the control group are similar to the abovementioned reports. However, selenium and melatonin supplementation (alone or in combination) for a one month suppressed MDA increase in AC and has reduced the levels close to control group levels. Results of previous studies indicating that both selenium and melatonin have suppressed MDA increase associated with ischemia-reperfusion support our findings [36]. In previous studies, selenium supplementation has been reported to suppress the increase in lipid peroxidation associated with ischemia-reperfusion, which emphasizes the role of selenium supplementation [30, 37, 38]. Studies investigating the effects of melatonin treatment in ischemia-reperfusion have shown associated reduction in organ damage. In the study by Erdem et al. [39], MDA was significantly increased by skeletal muscle injury that was developed with 2-hour ischemia by femoral artery clamping followed by approximately 1.5-hour reperfusion and MDA increase associated with I/R was reduced with melatonin treatment. Wang et al. [40] reported that melatonin administered parenterally 10 minutes before ischemia and 10 minutes after reperfusion significantly improved mitochondrial dysfunction associated with ischemia-reperfusion.

In our study, changes in MDA levels in AC were investigated as a marker of ischemic damage in an ocular ischemic syndrome after 1 month of oral selenium and melatonin treatment to allow for chronic evaluation. Our study has revealed similar results compared to the abovementioned studies in terms of significantly increased MDA values in AC associated with ischemia-reperfusion as a marker of damage. Significant suppression of MDA increase in AC detected with 1 month of selenium and melatonin supplementation indicates the protective effects of these two substances against ischemic damage. These findings are also similar to the previous reports [41].

In our study, glutathione levels were also measured as a marker of the antioxidant system. GSH values in AC were found lower in the OIS group compared to other groups. Selenium and melatonin supplementation, alone or in combination, resulted in significant increase in GSH levels. Previous studies have reported that selenium supplementation provided protection against oxidative damage by increasing glutathione peroxidase (GSH) [30, 38, 42].

In our study, protection against oxidant damage was obtained by selenium supplementation for 1 month (1.5 mg/kg, twice daily), as measured by increased GSH levels in AC. Similarly, 1 month of melatonin supplementation has also resulted in significantly increased AC GSH levels. Melatonin is a considerably potent antioxidant and this effect has been established in various ischemia-reperfusion studies. The study by Yilmaz et al. [43] has shown that kidney tissue was protected against oxidative stress caused by ischemia-reperfusion. Melatonin has been reported to provide this effect through increased GSH levels in heart tissue by Liu et al. [44], in liver by Deng et al. [45], and in spinal cord injuries by Aydemir et al. [46]. Our finding indicating increased GSH with melatonin treatment is supported by the above-mentioned studies. However, it should be noted that highest AC GSH levels were found in Group 5 (selenium + melatonin). Therefore, it was established that combined treatment was more effective for providing increased AC GSH values compared to either treatment alone.

The aim of this presented study was to determine the effects of selenium and melatonin administered for one month alone or in combination on lipid peroxidation in patients with ocular ischemic syndrome. Since there is not much work related to this topic in the literature, we think that this study can lead to many new studies related to the subject.

The limitation of our study is that the number of patients who participated in the study is very low. Patient population can be extended in other studies and also we did not assess other biomarkers of oxidative stress such as catalase, glutathione peroxidase, and 8-hydroxy-deoxy guanosine. We believe that making new studies in these biomarkers will make new contributions to the literature.

## 5. Conclusion

In conclusion, in OIS patients, MDA levels were found significantly increased in AC, as a marker of oxidant damage. On the other hand, selenium and/or melatonin supplemented alone or in combination for 1 month was shown to provide protection by resulting in increased levels of the antioxidant GSH.

## Figures and Tables

**Table 1:** MDA values in anterior chamber.

Groups	MDA in AC (nmol/ml)	*p* value (*p* < 0.001)
Control group (*n* = 7)	0.04 ± 0.01	0.193
Ischemia group (*n* = 7)	0.45 ± 0.08	0.000003^*∗*^
Selenium + ischemia group (*n* = 7)	0.06 ± 0.03	0.496
Melatonin + ischemia group (*n* = 7)	0.08 ± 0.05	0.768
Selenium + melatonin + ischemia group (*n* = 7)	0.10 ± 0.06	0.323

^*∗*^Statistically significant (*p* < 0.0011 for MDA).

**Table 2:** GSH values in anterior chamber.

Groups	GSH in AC (mg/dl)	*p* value (*p* < 0.001)
Control group (*n* = 7)	0.42 ± 0.05	0.231
Ischemia group (*n* = 7)	0.15 ± 0.01	0.000081^*∗*^
Selenium + ischemia group (*n* = 7)	0.52 ± 0.03	0.602
Melatonin + ischemia group (*n* = 7)	0.48 ± 0.08	0.439
Selenium + melatonin + ischemia group (*n* = 7)	0.59 ± 0.02	0.561

^*∗*^Statistically significant (*p* < 0.001 for GSH).

## Data Availability

The data used to support the findings of this study have been deposited in ClinicalTrials.gov (NCT04005222), and the data used to support the findings of this study are included within the article.
